# Long-Term Preclinical Evaluation of a Permanent Stent Developed for the Human Eustachian Tube

**DOI:** 10.3390/bioengineering11080755

**Published:** 2024-07-25

**Authors:** Katharina Schmitt, Malena Timm, Philipp Krüger, Niels Oppel, Alexandra Napp, Friederike Pohl, Robert Schuon, Andreas Kampmann, Lisa Kötter, Marion Bankstahl, Thomas Lenarz, Tobias Stein, Gerrit Paasche

**Affiliations:** 1 Department of Otorhinolaryngology, Hannover Medical School, Carl-Neuberg-Str. 1, 30625 Hannover, Germany; schmitt.katharina@mh-hannover.de (K.S.); timm.malena@mh-hannover.de (M.T.); schuon.robert@mh-hannover.de (R.S.); koetter.lisa@mh-hannover.de (L.K.); lenarz.thomas@mh-hannover.de (T.L.); 2 bess pro GmbH, Gustav-Krone-Str. 7, 14167 Berlin, Germany; p.krueger@besspro.eu (P.K.); t.stein@bessgroup.com (T.S.); 3 Department of Cranio-Maxillo-Facial Surgery, Hannover Medical School, Carl-Neuberg-Str. 1, 30625 Hannover, Germany; kampmann.andreas@mh-hannover.de; 4 Institute for Laboratory Animal Science and Central Animal Facility, Hannover Medical School, Carl-Neuberg-Str. 1, 30625 Hannover, Germany; bankstahl.marion@mh-hannover.de; 5 Institute of Pharmacology and Toxicology, Department of Biological Sciences and Pathobiology, University of Veterinary Medicine Vienna, 1210 Wien, Austria; 6 Cluster of Excellence Hearing4all, Hannover Medical School, Carl-Neuberg-Str. 1, 30625 Hannover, Germany

**Keywords:** nitinol stent, Eustachian tube, tympanometry, histology, CBCT, ETD treatment, self-expanding metallic stent, in vivo, sheep

## Abstract

The Eustachian tube (ET) is a bottleneck when it comes to middle ear (ME) health. If its function is impaired, this can lead to serious consequences for the patient, such as hearing problems or deafness. Therefore, this study investigated a tapered nitinol stent (3–5 mm × 14 mm) for the human ET as a potential new permanent treatment for chronic Eustachian tube dysfunction (ETD) and thus ME ventilation disorders. The self-expanding stent was inserted unilaterally into the ET of 24 sheep with observation periods of 3, 6, and 12 months. Local tissue effects and the safety of the stent insertion were analyzed based on regular endoscopic checks, weekly tympanometry measurements, final imaging, and histological examinations. The animals showed no stent-related health restrictions. However, the individual anatomy and stenting procedure had an influence on the results. The tissue reaction in the endoscopic examinations was mild even though no concomitant antibiotics were administered. After all three monitoring periods, stented ETs had a significantly larger ET lumen than the non-stented contralateral ETs. However, tissue growth was detected in the stent. Overall, the first long-term study on an ET stent showed that the tapered ET stent could be a promising treatment option for ETD.

## 1. Introduction

This study aimed to test newly developed nitinol stents for the human Eustachian tube (ET) in sheep. A previous study showed that the ET of sheep resembles the anatomy of humans and therefore medical devices developed for humans can be tested in this species [1]. The ET is a narrow cavity that consists of a cartilaginous and a bony part [2]. It connects the middle ear with the pharynx. For this small luminal organ, literature describes the total length in adults to be up to 40 mm [3,4,5]. The tube opens through a complex interaction of muscles, fascia, ligaments, and the Ostmann fat pad [2,6]. Its lumen is lined with pseudostratified columnar epithelium of the ciliary type, which enables clearance of secretions [5,7]. 

The ET has three main functions. It drains the middle ear by transporting mucus through ciliary activity to the pharyngeal orifice. It ventilates the middle ear (ME) and equilibrates the pressure. It prevents pathogens and tones from ascending to the ME. A disruption of the functions could lead to otitis media (OM) or autophony [3,8]. The inability to perform at least one of the mentioned functions describes the pathology of Eustachian tube dysfunction (ETD) [9]. ETD can result in mucus accumulation and lead to otitis media, pathological middle ear pressure or, in case of a patulous ET, to autophony. Thereby, a higher risk for hearing disabilities and in extreme cases deafness appears obvious. However, ETD can also be caused by OM, tumors, infections, allergies, and nerval or muscular problems [10,11]. 

Various approaches to treat ETD were described. Current ETD interventions are, for example, balloon Eustachian tuboplasty (BET) [12,13], microdebrider Eustachian tuboplasty [14], laser Eustachian tuboplasty [15,16], nasal douching, and topical decongestants [17]. There is a lack of long-term ETD treatment data, but BET seems to be the most promising option up to now [18]. BET is described as a safe and helpful treatment in ETD and can improve tympanogram values [12,19,20]. However, in addition to many positive reports, there are also publications that show less favorable outcomes. In one systematic review [21], for example, BET was found to result in a regularly ventilated middle ear (type A tympanogram) in only 86 of 141 ears (61%). There are also patients who do not benefit from BET even after repeated application [22].

Development of new treatment options for ETD such as stents for the ET could potentially help to overcome current limitations. Stent prototypes have already been implanted in vivo in the ETs of rabbits [23], chinchillas [24], cats [25], pigs [26], and sheep [27]. Furthermore, stent implantations in human cadavers were performed. One study used a balloon expendable cobalt chromium (CoCr) stent prototype in six cadavers and compared two different diameters [28]. Another cadaver study tested ET stent prototypes made of different materials, e.g., nitinol (NiTi), CoCr, and poly-L-lactic acid (PLLA) [29]. 

In a few studies, patients even received support for the ET lumen after different interventions. Angiocatheters were used provisionally serving as ET stents in humans after surgical interventions, e.g., ET reconstruction [30] and nasopharyngectomy [31]. Angiocatheter placement in the ET was proven to be more successful than tympanostomy tube placement in the prevention of otitis media with effusion (OME) after nasopharyngectomy surgery [31]. However, it was necessary to regularly remove crusts from the end of the catheter, which protruded into the pharynx. In one case, a ureteral stent was cut to size and implanted after recanalization of an obliterated ET to maintain the neo-lumen [32]. These approaches emphasize the need for stents for the ET. As shown by Pohl et al. [27] using cardiovascular stents in vivo in sheep, the shape and length of the stents need adjustments to fit the ET. This was also confirmed by Kim et al. after CoCr stent placement in the ETs of three pigs [26].

First approaches with shape-adapted stents inserted into the ET were conducted recently [33]. A tapered self-expanding metallic stent (SEMS) prototype was compared to a tubular SEMS in a porcine in vivo model with three animals each [33]. Both stents had a length of 16 mm and protruded into the pharyngeal lumen. The tapered stent had less formation of tissue hyperplasia compared to the tubular design.

Based on the human cadaver study [29], shape-adapted stents made from nitinol were developed for implantation in the human ET. These permanent stents were now investigated in a preclinical study in sheep regarding their tolerability, behavior, and safety with observation periods of up to one year.

## 2. Materials and Methods

### 2.1. The Ethical Statement and Animals

The in vivo experiments were conducted in 24 adult, female German black-headed meat sheep in accordance with the German Animal Welfare Law and the Directive 2010/63/EU. The State Office for Consumer Protection and Food Safety, Dept. of Animal Welfare, of Lower Saxony, Germany, approved this study under the number 19/3255. The sheep were obtained via the Central Animal Facility (CAF) of Hannover Medical School from a farm, where they were already used to direct contact with humans, and housed in the CAF. Animals were on average 4 years old (min: 2 years; max: 6 years) and weighted 80.20 kg ± 11.66 kg (mean ± SD) on the first day at the CAF. After arrival, the animals were divided into small flocks (2–10 sheep) and kept in straw-covered stables with outdoor climate and continuous access to tap water and hay. The health status of the sheep was examined by a veterinarian on the day of arrival and checked regularly during the course of the study. After arrival at the CAF, the sheep were quarantined, and they were assigned to one of the three study groups by drawing lots. Sheep were trained for 4 weeks to tolerate handling and tympanometry measurements (see Section 2.5). Training was supported by using proportions of the daily fed complimentary feed pellets (V5103-000, complimentary feed for sheep and goats, 4 mm pellet, ssniff-Spezialdiäten GmbH, Soest, Germany). The experiments were conducted in compliance with the legal directives for accommodation, care, and usage of experimental animals.

### 2.2. Eustachian Tube Stent and Application Tool

**Figure 1:**
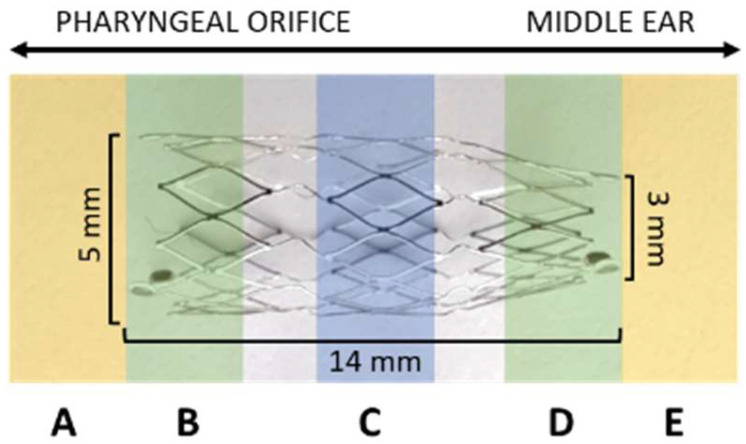
Eustachian tube stent and its dimensions [mm]. Alignment of the stent to the middle ear and the pharyngeal ET orifice (arrows) is indicated. The stent is displayed with defined histological cutting sections (**A**–**E**).

Stents and application tools [34] were provided by bess medizintechnik GmbH (Berlin, Germany). The stent (Figure 1) was made of a nickel and titanium shape memory alloy (nitinol, NiTi) with 3 tantalum X-ray markers at each end. It had a length of 12 mm without and 14 mm with the markers. The diameter of the stent decreased from 5 mm to 3 mm, which creates its tapered shape to adjust to the anatomy of the ET. It is a SEMS manufactured to fully deploy at physiological body temperature. 

**Figure 2:**
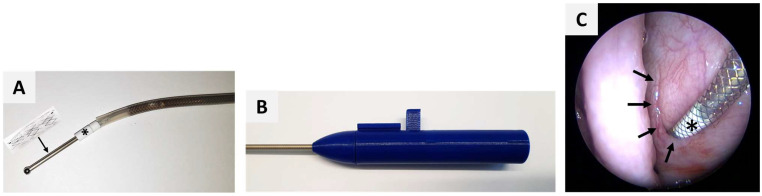
Application tool with tip (**A**) and handle (**B**) forwarded (**C**) into the crescent-shaped pharyngeal ET opening (arrows) with a white depth marker (*) to control insertion depth.

The application tool had a length of 30 cm to fit the anatomy of sheep. It was sterilizable and had a 3D-printed handle and a bendable metallic tip with a sphere (approximately 2 mm) at its tip for atraumatic usage. The application tool came loaded with the stent crimped on the metallic tip covered by a transparent outer sheath. The tapered end of the NiTi stent was directed to the metallic sphere of the tip and therefore to the middle ear when the tool was inserted. A white depth marker allowed controlling the insertion depth (Figure 2). The tool was accompanied by a template for defined bending of the tip to adjust to the specific anatomy of each animal. Angles could be set between 0° and 45°.

### 2.3. Study Design and Schedule

The study design was based on previous sheep experiments [27,35]. Three groups with *n* = 8 each were established. Every sheep was implanted single-sided, and the contralateral ET acted as control. Sides were chosen by randomization prior to the stent insertion. Follow-up periods were 3 months (3M group) after implantation, and 6 and 12 months (6M group and 12M group), respectively. 

All animals underwent four endoscopic procedures under general anesthesia (GA). Food was withheld for 18–24 h before the procedures, but the animals had free access to water. GA was induced with intravenous (i.v.) propofol (5–10 mg/kg i.v.; Narcofol^®^ 10 mg/mL, CP-Pharma GmbH, Burgdorf, Germany) administration after previous sedation (0.2 mg/kg i.v.; Midazolam-ratiopharm 15 mg/3 mL, ratiopharm GmbH, Ulm, Germany). This was followed by inserting a gastric tube into the rumen to prevent gastric tympany and aspiration of gastric juices, and orotracheal intubation to maintain anesthesia with isoflurane (1.5–2.0% end-tidal concentration; Isoflurane CP 1 mL/mL, CP-Pharma) in 1:1 oxygen and air (average flow rates of 1 L/min). Peri-operative analgesia was secured with carprofen (2 mg/kg i.v., once following induction of GA; Carprosol^®^ 50 mg/mL, CP-Pharma). The sheep were placed on an operating table in sternal recumbency and either kept in spontaneous breathing or artificially ventilated if necessary. Throughout GA, lactated Ringer’s solution was administered (5–10 mL/kg/h, i.v., Ringer-Lactat-Lösung ad us. vet^®^; WDT, Garbsen, Germany). Blood oxygen saturation, heart rate, rectal temperature, and respiratory rate were monitored continuously. During the first GA, cleaning of the external auditory canal (EAC), and inspection of the tympanic membrane and the pharyngeal orifices of the Eustachian tubes, were performed. At the second GA, endoscopic inspection and unilateral stent placement were performed. Further controls under GA with endoscopic examinations of the pharyngeal ET openings were performed at half-time of the study period for each group (3M at 1.5 months; 6M at 3 months, and 12M at 6 months) and at the end of the defined test period for each animal. Following GA, the sheep were returned to their barn and kept under close observation until they were able to stand and eat.

The sheep were euthanized at the end of the final GA through an excessive intravenous amount of pentobarbital (Release^®^, 300 mg/mL, WDT). After separation from the body, the head was put on ice, and directly forwarded to cone-beam computed tomography (CBCT, see Section 2.7). Subsequently, the animals’ heads were dissected (FK 23 bone saw, Bizerba, Balingen, Germany), and the ETs were prepared for histology (see Section 2.8).

### 2.4. Health Score

The health of the animals was monitored every 2 to 3 days, and an established health score [36] was applied. After the stent insertion, animals were scored every day for one week. This secured and facilitated the detection of health abnormalities with special emphasis on the upper and lower respiratory tract. In addition, the score was used to document the behavior of the animals and, therefore, any discomfort that might be caused by the stent would influence the score. Evaluated categories were vocalization, activity, feed/water intake, behavior/facial expression, breathing rate, and nasal discharge.

### 2.5. Tympanometry 

Throughout the study, the middle ear pressure was monitored by tympanometry as described in Pohl et al. [35]. The middle ear status was assessed before stent insertion to ensure a physiological ET function. After stent insertion, measurements were conducted weekly by using a Madsen OTOflex100 tympanometer (GN Otometrics, Münster, Germany) calibrated to a custom-made adapter for the sheep ear. Both ears were measured three times in a row to ensure valid data as small movements of the sheep can affect the measured pressure curve. The data was then transferred to and evaluated with the appropriate software (OTOsuite^®^, version 4.84, GN Otometrics). The received curves were categorized according to the defined curve types in sheep [35]. Curve types A/An were considered physiologic, type B was flat or a curve with only a small amplitude (possible OME, tympanic membrane disruption, or disturbance during measurement), and type C was similar to type A but with a shift of the maximum to negative pressure by more than 100 daPA indicating ETD. 

### 2.6. Endoscopy under GA

All endoscopic examinations including the stent insertion were conducted under GA (see Section 2.3).

#### 2.6.1. Inspection of the External Auditory Canals

Prior to the endoscopic examination of the tympanic membrane, the EAC was cleansed from ear wax with an ear forceps (221100 Hartmann EAR Forceps with very fine serrated jaw 1 × 4.5 mm, WK length 8 cm; KARL STORZ, Tuttlingen, Germany). If needed, the EAC was cleansed with Otodine^®^ (active substances: chlorhexidine digluconate, Tris-EDTA, pH 8.0; aniMedica (LIVISTO), Senden-Bösensell, Germany). This process was conducted under every GA if necessary. From the first GA until the end of the experiment, Otodine was applied into both EACs once a week 2–3 days prior to tympanometry, to maintain clean EACs throughout the experiment.

#### 2.6.2. Inspection of the Pharyngeal Opening of the ET

To prepare for every transnasal endoscopy, longitudinal swabs containing a mixture of xylomethazolinhydrochlorid (Otriven^®^, 0.1%, 10 mL, 1 mg/mL, GlaxoSmithKline consumer healthcare GmbH & Co. KG, Munich, Germany) for decongestion and 1 phial lidocaine hydrochloride 1 H_2_O (Xylocitin^®^-loc 2%, 5 mL, Mibe GmbH Arzneimittel, Brehna, Germany) for local anesthesia were inserted through the nostrils into both nasal cavities and left in place for 5 min. Video imaging was conducted with a rigid endoscope (HOPKINS^®^ 70°, 4 mm diameter, 30 cm length, KARL STORZ SE & CO. KG, Tuttlingen Germany) and enabled a full view of the ET opening during the stenting process and the follow-up examinations. The endoscope was connected to a camera head (telecam PAL, KARL STORZ) and a fiber optic light cable and attached to a Tele Pack Vet X LED RP 100 video system (KARL STORZ).

The pharyngeal ET opening was categorized for its degree of opening. A distinction was made between closed, slightly open, distinctively open, and severely open. During the inspection of the ET orifice, secretion, if present, was evaluated. Amount and character were assessed for both sides. The amount of the ET discharge was scored as not present (score 0), small (score 1), medium (score 2), or excessive (score 3). In addition, the character of the secretion was evaluated and scored from not present (score 0), serous (score 1), sero-mucous (score 2), or mucous (score 3). Since there were only few secretions in the entire period of the study and secretion was also detected at control sides, the classification was averaged as normal (scores 0 and 1) or abnormal (scores 2 and 3) regarding the amount and character.

#### 2.6.3. Stent Insertion

Stent insertion was conducted during the 2nd GA. The tool was introduced through the *Meatus nasi ventralis* of the nasal cavity up to the pharyngeal orifice of the desired ET. The other nasal cavity was used for the endoscope, which enabled a full view of the ET opening during the stent insertion process. The tool was forwarded into the ET lumen (Figure 2). The stent was released passively into the ET lumen by retracting the outer sheath of the tool around the stent, which then allowed the stent to deploy. After that, the tool was slowly and carefully withdrawn from the ET lumen.

Per definition, the white depth marker of the application tool should be fully inserted into the ET lumen and conclude with the ET opening, before the stent was released. Three depth positions were distinguished in the video endoscopy: 100% indicating a fully inserted depth marker; >50% if the vast majority was in the lumen; and <50% if the marker was mostly visible outside the ET lumen.

### 2.7. Imaging

CBCT scans were taken of the full head of each sheep and, additionally, the region of the implanted ET (mode: CT, 360°; device settings: HiFi and HiRes imaging; 170 × 120 and 60 × 60, respectively) using a 3D Accuitomo 170 (J. Morita Mfg. Corp., Kyoto, Japan). The data were saved as DICOM files and analyzed using the software 3D Slicer (https://www.slicer.org/; version 4.11.20210226; accessed on 17 March 2022) [37].

The total length of every stent was measured and its diameters of the cross-sections at both ends (d3—facing the pharyngeal ET opening; d1—facing the ET isthmus) and the center (d2) were accessed. The locations of the diameters corresponded to the histological sections D, C, and B (compare Figure 1). At first, the largest diameter was measured for each section (d1–d3) and, from this, the orthogonal diameter. As such, the shape of the stent was evaluated.

Additionally, the middle ear status was analyzed and categorized into free and therefore ventilated, partially filled, or completely filled with fluid and/or tissue. This provided information on the possible occurrence of middle ear effusion, which could lead to type B or C tympanograms.

### 2.8. Histology

**Figure 3:**
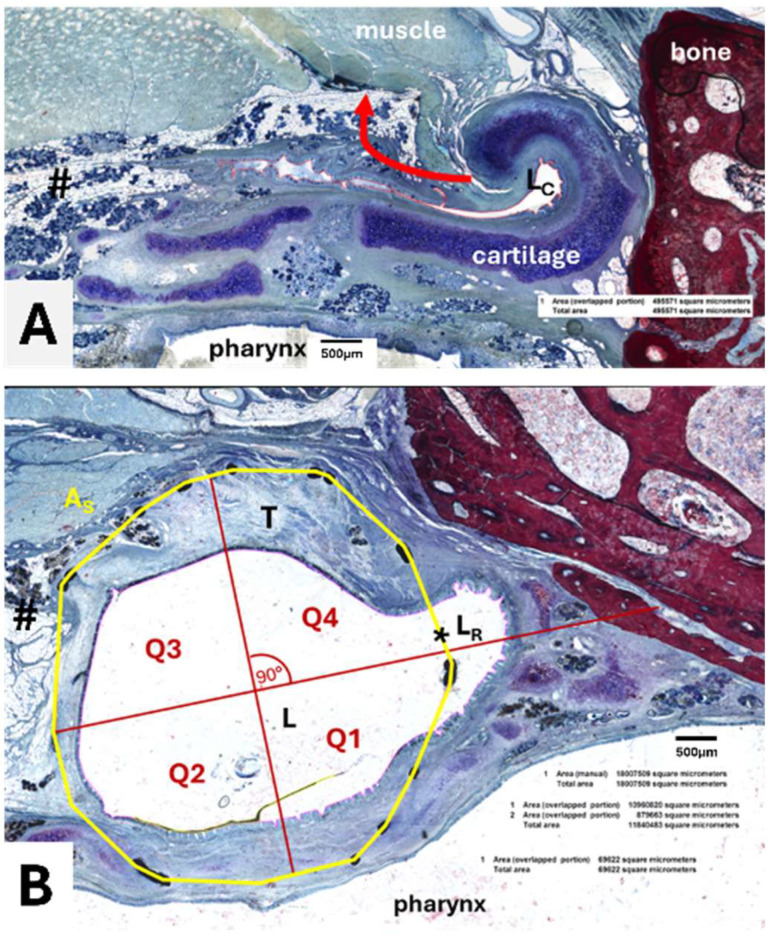
Histological sections of the Eustachian tube. (**A**) Cross-sectional view of a control ET with an arrow indicating the movement during pressure equalization. Lumen of control (L_C_, surrounded by a red line), Ostmann fat pad, and glandular tissue (#) are indicated. (**B**) Cross-sectional view of cutting section C with area of the stent (A_s_, yellow line), stent lumen (L), ingrown tissue (T), residual tubal lumen (L_R_), and the quadrants (Q1–Q4). The dividing lines of the quadrants were defined by a first line from the center of the stent to the center of the intersection (*) between the stent and the residual lumen and a subsequent orthogonal line.

After preparation, the tissue containing the ET was fixed with 3.5% formalin (C. Roth, Karlsruhe, Germany) at pH 7.4 for 3 weeks. Formalin was exchanged twice per week. The specimens were dehydrated in an increasing ethanol protocol for 10 days (70%, 80%, 90%, 100%; Merck, Darmstadt, Germany). After dehydration, the samples were infiltrated with methyl methacrylate under vacuum and polymerized in a water bath for 2 to 4 days (depending on the status of polymerization) with an increasing temperature (up to 37 °C) for the exothermal reaction. The size of the sample was reduced by trimming any excess material. Samples were sliced into ET cross-sections (thickness approximately 45 µm) by using a saw microtome (Leica-SP1600^®^, Leica Biosystems Nussloch GmbH, Nussloch, Germany). Three consecutive slices were taken from the three defined cutting sections of the stent (B, C, and D) (Figure 1). Additional slices were taken from the regions in front of and behind the stent, identified as sections A and E in Figure 1. The slices dried over night at 37 °C in a metal press followed by staining with methylene blue (Löffler’s methylene blue solution; Merck) and Alizarin red (Alizarin Red staining solution; Merck) before mounting them on the microscopic slide with Entellan (Entellan^®^ Neu; Merck). Histological imaging and analysis were conducted using a digital microscope at 2× and 4× magnification (BZ-9000^®^ and BZ-II-Analyzer program; KEYENCE, Osaka, Japan). Several images of the microscopic slides were taken and merged to one image (Figure 3).

For analysis, the area of the stent (A_S_), the lumen in the stent (L), the area of this lumen taken by secretion (S), and the area of the stent occupied by granulation tissue (T) were determined (Figure 3). The residual ET lumen (L_R_) apart from the stent lumen and its secretion S_R_ were also determined. The total ET lumen (L_T_) was defined as the sum of L and L_R_. For the control side the ET lumen (L_C_) and secretion (S_C_) were measured (Figure 3A). In addition, the stent area was subdivided into four quadrants of a circle (Figure 3B) approximating the stent circumference to distinguish the status of the epithelium at different parts of the ET. The epithelium was rated intact (>80% intact; score 1), intact but incomplete (>20–80%, score 2), or not intact (<20%, score 3) for each quadrant. In addition, a score 4 was applied for quadrants with no epithelium, e.g., the quadrant was completely filled with tissue. The epithelium of the control ET was also classified and assigned to one of the categories.

### 2.9. Statistics

Sample size was calculated with G*Power [38,39]. If not otherwise stated, data are expressed as mean ± standard deviation. A *p*-value below 0.05 was considered to indicate significant differences. Analysis was conducted using GraphPad Prism 9 (GraphPad Software Inc., La Jolla, CA, USA). Average values of three microscopic slices per cutting section were determined before calculating group mean values or mean values for entire stents. Animals 3M-4, 6M-1, 6M-2, 6M-8, 12M-3, and 12M-4 had to be excluded from statistical analysis of histological data for different reasons (see Section 3.5). Prior to the analyses, the data were tested for normal distribution using the D’Agostino–Pearson test (if *n* = 8) or the Shapiro–Wilk test if *n* < 8 values. Statistical evaluation for differences between the groups was performed using one-way ANOVA. The prerequisite for this analysis was normal distribution of the residuals and variance homogeneity (Brown–Forsythe test). The Kruskal–Wallis test was used if normal distribution was not given. Additionally, post hoc tests for multiple comparisons between the groups were carried out (Tukey’s or Dunn’s multiple comparisons test). Moreover, the stent lumen in comparison with the control for each group was evaluated using a paired *t*-test or Wilcoxon’s matched pairs signed rank test, depending on the result of the normality test. The quadrants were compared to their controls conducting the Friedman test. In case non-parametric tests had to be used, the test is indicated accordingly.

## 3. Results

### 3.1. General Remarks

Small adjustments of the initial study plan had to be made due to individual influences by the animals. One animal did not meet the inclusion criteria as it showed type B tympanograms on both sides. It was replaced by another sheep. Additionally, a change of the randomized stent side was necessary for 3M-4 and 6M-3. Animal 3M-4 had constant type B tympanograms on the left ear prior to implantation. Therefore, the stent was inserted into the right ET. For case 6M-3, issues to get the tool into the left ET resulted in stent placement into the other ET.

Throughout the experiment, animals were healthy, showed no head tilting or shaking, and predominantly scored 0 in all categories of the health score. However, two animals had deviations in the scoring. Animal 6M-8 scored with 0.5 for one day because of an elevated respiratory rate. The animal was diagnosed with hypocalcemia after the first GA and recovered after treatment (15 mL/50 kg s.c., Calcitat S50^®^ ad us. vet, aniMedica (LIVISTO); 30 µg/kg s.c., Vitamin D_3_ 1.000.000 I.E.^®^ ad us. vet., 25 mg/mL, CP-Pharma; Vitamin B1 Hevert^®^, 157 mg Thiamin/2 mL, 10 mg/kg s.c., Hevert-Arzneimittel GmbH & Co. KG., Nußbaum, Germany), showing no problems after the following GAs. Animal 12M-3 had bloody, serous unilateral nasal discharge (left nostril) which generated a score 2 on two days in succession. On the following day, the animal had only serous nasal discharge and scored 1. This took place in week 10 after stent insertion into the right ET.

The results of the insertion process, endoscopy, tympanometry, CBCT, and histology are presented individually below. This is followed by a chapter that links the data from the different examinations (see Section 3.6).

### 3.2. Endoscopy

Endoscopy was used to not only observe stent insertion and control insertion depth but also to inspect pharyngeal orifices at half-time and final follow-ups, and the tympanic membranes.

Prior to the stent insertion, the tympanic membranes of the groups had no visible abnormalities for both ears except for animal 12M-2. This animal had a small defined area with altered mucosa before the stent insertion. This was also visible but smaller in the two follow-ups of 12M-2. The other animals (23/24) had physiologic tympanic membranes in the half-time follow-ups for the stent side and the control side. In the final follow-up, animal 12M-7 showed an uneven tympanic membrane with partially whitish tissue on the stent side. The remaining sheep (22/24) presented with normal tympanic membranes in the last endoscopy check for both sides.

In some cases, it was difficult to approach the ET and insert the tool at the matching angle to reach the full insertion depth. The angles of the application tools had to be adjusted individually. The final angles used for the tool tip varied from 0° to 45° (see Section 3.6). With *n* = 7, the most frequently used angle was 20°; the average angle was 24.6°.

When evaluating the video endoscopy, it was noticed that there were deviations in the stenting procedure. For three stent insertions (3M-4, 6M-1, 6M-2), the tool was advanced slightly while the outer sheath was withdrawn. In addition, a small plaque-like adhesion on the mucosa with no connection to both ET sides was detected in the rear, upper area of the left nasal cavity (anatomical region of *Concha nasalis media* and *Meatus nasi medius*) during GA of 12M-3 prior to the stent application (right side).

Insertion depth was assessed for every stent placement. The desired 100% insertion depth was fully achieved in 15/24 stent insertions. Despite adjusting the insertion angle, feelings of resistances during the tool insertion process resulted in insertion depths not fully reached. Slightly deviating (>50%) were four out of 24 positionings. However, five out of 24 depth markers could only be positioned into the ET lumen with less than 50% of its length (Table 1).

In other cases (animals 3M-2, 6M-8, 12M-6, and 12M-7), the stent was slightly pulled back in the direction of the ET orifice during removal of the tool after release of the stent. In the half-time and final checks, a perforation of the mucosa at the ET opening was detected for animal 6M-8. Stent struts were bulging into the pharynx lumen through the perforated mucosa.

**Figure 4:**
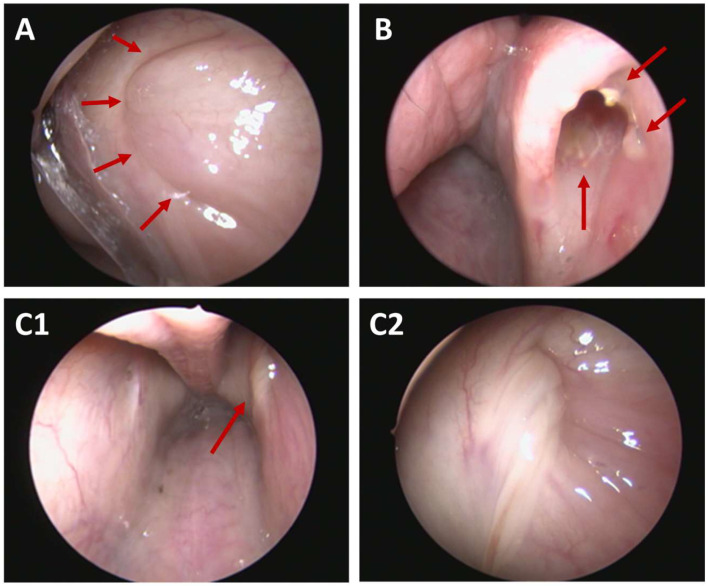
Endoscopic view of Eustachian tube orifices. (**A**) Closed left ET (arrows) with normal secretion (score 0) at 12 months post-stent insertion (12M-2). (**B**) Severely open ET with visible stent (arrows) at 1.5 months after stent was applied too close to the ET opening with insertion depth <50% (3M-7). (**C1,C2**) Abnormal secretion (arrow, score 3) in overview of both ET openings (**C1**) and in close-up of the left side (**C2**) at 3 months follow-up (6M-4).

Throughout the observation period, the ET orifices were analyzed in terms of their opening grades (Table 2). Before implantation, all ET openings including the control sides were closed (Figure 4A). Directly after the procedure, nine out of 24 stented ETs were closed, and the others varied from slightly to distinctively open. For two animals (3M-1; 12M-4), the opening was monitored as severely open, and the stent was visible in the ET lumen. For group 3M, cases 3M-1, 3M-3, and 3M-7 showed severely open ETs in the follow-ups. Animals 6M-3 and 6M-8 were categorized as severely open in the last follow-up. However, 6M-8 is the case with the fenestration of the mucosa.

In some cases, the stent was visible by endoscopy in terms of stent struts shimmering and/or perforating through the mucosa and/or the stent was visible through the ET opening (Figure 4B). In a total of nine out of 24 cases the stent was visible during half-time and/or final follow-ups. In seven out of nine cases with visible stents at the half-time follow-up, these were also visible at the final follow-up (including the fenestration of case 6M-8). Only one stent was visible at the half-time check but not in the final check. Another one was only detectable in the final follow-up.

Throughout the experiment, only few stented ETs presented with mucous deposits adhering to the mucosal layer of the orifice, or if present, the visible stent struts. At the half-time follow-up, five cases (3M: *n* = 3; 6M: *n* = 1; 12M: *n* = 1) had deposits at the orifice. The same five animals plus one other case of group 3M showed deposits in the final endoscopy check (detailed overview on affected animals is provided in Section 3.6). No control side had deposits at the ET orifice, neither at the half-time nor at the final follow-up.

In addition, the secretion from the ET openings was analyzed. All ETs including the control sides had normal secretion (score 0–1) in terms of amount and character prior to the stent insertion. Throughout the experiment, few deviations were detected during the follow-ups (compare Figure 4(C1,C2)).

For all three groups (3M, 6M, and 12M), control orifices showed a normal amount of secretion at all follow-up appointments (score 0–1). The stent sides of the 3-month group showed two out of eight ET openings with abnormal secretion (score 2–3) at half-time, whereas eight out of eight presented as normal in the final follow-up. The stented sides of group 6M had four out of eight abnormal ET discharges in the first, and three out of eight in the final, follow-up. The stented ETs of the 12-month group had two out of eight ETs with abnormal discharge at both endoscopic examinations.

The character of the ET discharge was classified as normal in the control sides of all three groups for every checkup (score 0–1). For the stent sides, the discharge was characterized as abnormal (score 2–3) in some cases at the first follow-up (3M: three out of eight abnormal; 6M: three out of eight abnormal, 12M: four out of eight abnormal) and the last follow-up (3M: four out of eight abnormal; 6M: five out of eight abnormal; 12M: three out of eight abnormal).

### 3.3. Tympanometry

**Figure A1:**
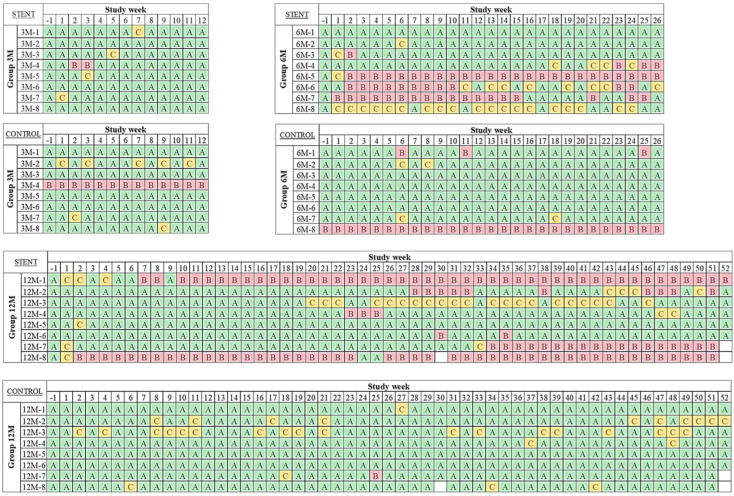
The first valid measurement of each week was included. Type A (green), type B (red), and type C (yellow). For incomplete measurements (white boxes) of animals 12M-7 (*n* = 1) and 12M-8 (*n* = 2), the values from the previous week were assumed.

Tympanometry was conducted weekly without any major difficulties. All animals (24/24) had physiological middle ear pressure with determined type A tympanograms for the stent sides prior to the stent insertion. For the control side, 22/24 animals had type A tympanograms before stent insertion. Animals 3M-4 and 6M-8 had type B tympanograms on the control ears throughout the observation period (compare Appendix A, Figure A1). Sporadic restrictions of the ME ventilation occurred on the control ears in every group and most animals. A total of 11/24 animals showed recurring type C curves on the control ears and three out of 24 were measured with sporadic type B curves.

Group 3M showed mostly physiologic tympanograms at the stented side. Only few sporadic non-physiological tympanograms (4× type C; 2× type B) occurred over the study period. All animals of this group (eight out of eight) finished the experiment with normal type A tympanograms for the stent side.

The 6-month group had noticeable deviations in the tympanograms for the stent side. Animal 6M-5 had constant type B tympanograms since the second week of the trial. Animals 6M-6 and 6M-7 showed frequent B types and 6M-8 was measured with recurring phases of C types for the stented side. However, at the end of the 6-month period, five out of eight sheep showed physiological type A tympanograms, two out of eight type B, and one out of eight type C.

Group 12M also showed individual changes during the tympanometry. Two animals were measured with constant type B tympanograms throughout the study period on the treated side (12M-1; 12M-8). Animal 12M-7 showed constant type B tympanograms from the 34th week up to the end. For this group again, five out of eight animals finished the experiment with physiological type A tympanograms whereas three out of eight concluded with a B-type tympanogram. Detailed results for individual animals are provided in Appendix A, Figure A1.

On average, all groups and both sides showed predominantly physiological measurements (Table 3). More than 80% of measurements on the control sides resulted in physiological type A curves. On the stented side, this amount was only achieved in group 3M. It should be noted that animal 12M-3 was conspicuous, with repeated audible swallowing prior to the tympanometry which was subsequently recorded in sporadic measurements with negative middle ear pressure (type C) for both sides (compare Appendix A, Figure A1).

**Figure 5:**
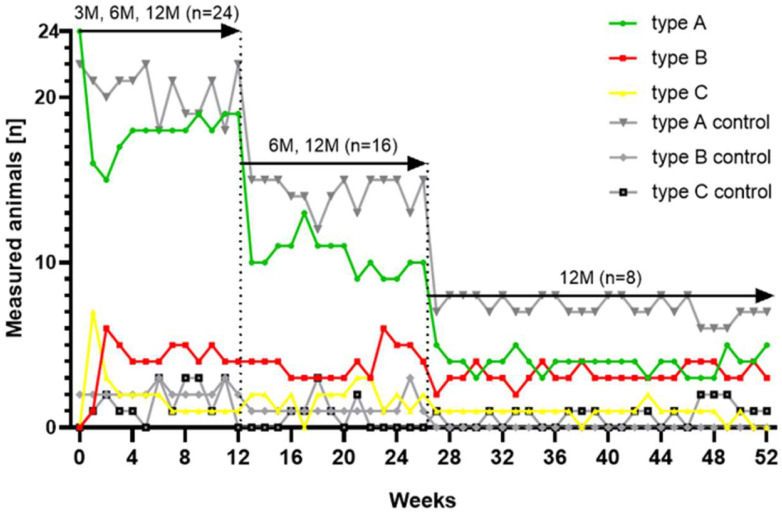
Distribution of tympanogram types for each study week and all cases (*n* = 24) associated with the color scheme of the individual results (Appendix A, Figure A1).

Figure 5 summarizes the time course of the results of the tympanometry. In the first 3 months, 24 animals contributed, then 16 animals, and in the last 6 months only eight animals contributed. On the stent side, the middle ear pressure shifted in some animals into a negative pressure range shortly after the stent insertion (curve type C), followed by a peak of type B tympanograms a week later. The increase in measured type B tympanograms in week 2 was accompanied by a decrease in type C tympanograms. Even though there is variation in the tympanometry results over time in most animals on the stented side and to a minor extent also on the control side, no overall increase or decrease in the number of ears with disturbed ME ventilation was observed throughout the observation period of 12 months.

### 3.4. Imaging

**Figure 6:**
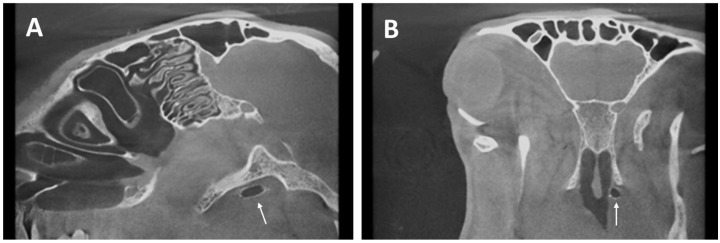
CBCT of 3M-7. Ventilated stent in longitudinal (**A**) and cross-section (**B**) of the sheep skull indicated by arrows.

**Figure 7:**
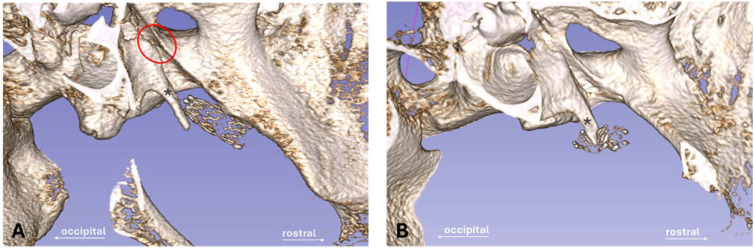
Dense and bony components of the head shown with 3D Slicer. (**A**) Stent (3M-5) is directing to the channel-shaped isthmus area (red circle) with proximity to the *Proc. muscularis* (*). (**B**) Compressed stent of animal 6M-2 winding around the *Proc. muscularis* (*) after incorrect stent insertion procedure.

CBCT data showed that 23/24 inserted stents could be located (Figure 6) at the end of the study. However, no stent was found in 12M-3. The sheep was regularly stented (right side) and followed the study protocol as planned. In this animal, a decline of the conchae nasalis of the left nasal cavity was found in CBCT. Imaging proved that 20/24 inserted stents were found to be positioned in the ET. However, three stents (3M-4, 6M-1, and 6M-2) were not positioned as desired. The stents were found mostly in the surrounding tissue of the ET and were not directed towards the middle ear. The stent of 3M-4 was positioned orthogonal to the ET direction and the stent of 6M-1 was in the surrounding ET tissue. CBCT also revealed that the stent of 6M-2 had an abnormal, compressed shape (Figure 7B).

No stent fractures were detected in CBCT. Stent location was visually determined in relation to the individual bony anatomy of the animals based on the *Proc. muscularis* (Figure 7) and the channel-like bony portion of the ET [40]. All stents were located close or next to the *Proc. muscularis* and no stent was positioned in the bony ET part, meaning that all 20 stents in the ET were positioned as deep as anatomically possible in the cartilaginous part of the ET. However, these anatomical landmarks varied in shape and length.

Additionally, the length of the stents was approximated by measuring the distances from X-ray marker to X-ray marker. The values were 13.5 ± 0.5 mm, 13.3 ± 0.2 mm (without the compressed stent 6M-2), and 13.4 ± 0.3 mm for the 3M, 6M, and 12M groups, respectively. No difference in stent length was found between the groups (Kruskal–Wallis test; *p* = 0.1081).

Furthermore, the diameters (d1–d3) of the stents for the three cross-sections were analyzed (Table 4). Data showed that 22/23 of the remaining stents fully deployed to its tubular and tapered shape, except for case 6M-2, which was tangled around the *Proc. muscularis*. The largest diameters of each group for cross-sections d2 (center of stent) were 5.1 ± 0.1 mm (group 3M), 5.1 ± 0.2 mm (group 6M), and 5.1 ± 0.2 mm (group 12M). The corresponding orthogonal diameters of the cross-section d2 were 4.9 ± 0.1 mm (group 3M), 4.9 ± 0.1 mm (group 6M, without 6M-2), and 4.9 ± 0.2 mm (group 12M). The comparisons of the respective largest and orthogonal diameters of d1 (*p* = 0.9398; *p* = 0.2087), d2 (*p* = 0.7478; *p* = 0.5251), and d3 (*p* = 0.1606; *p* = 0.1342) using the Kruskal–Wallis test showed that there were no significant differences between the groups.

In addition, the middle ear status was assessed with CBCT scans for all animals of each group (see below, Section 3.6). Typically, the middle ear appeared ventilated. Only in six out of 24 cases, it was filled partially or completely with fluid and/or tissue at the stented side. One animal (6M-8) was diagnosed with a filled ME on the control side.

### 3.5. Histology

Histological analysis was performed on stented and control ETs. The ET lumen and secretion of both sides were examined (Table 5). The stented sides of animals 12M-3 (stent was lost) and 12M-4 (stent was cut during preparation of the sample) had to be excluded. In addition, the misplaced stents (3M-4, 6M-1, and 6M-2) were also excluded from statistics. However, the analysis for these three cases confirmed an intact, unaffected ET and showed an existing lumen for the stent and control sides. This proved that the incorrect placement had not influenced the lumen. Each of these animals had individually similar values of L and L_C_ (3M-4: L = 1.00 mm^2^, L_C_ = 1.19 mm^2^; 6M-1: L = 0.21 mm^2^, L_C_ = 0.21 mm^2^; and 6M-2: L = 0.38 mm^2^, L_C_ = 0.50 mm^2^). Single blades of hay were found in the ET lumen of 3M-6 and 3M-7. These were protruding from the pharynx into the ET lumen and were found in the free lumen of the ET without any obvious damage to the epithelium. In some cases, the X-ray marker was visible in the histological cutting section.

Histological examination showed that the nitinol stents had predominantly grown into the surrounding tissue and the struts had been overgrown by the ET epithelium. The stent supported the ET and maintained a lumen that was larger than in untreated ETs (compare Figure 3A,B). The lumen of the control ETs (L_C_) was comparable between the groups with no significant difference (Kruskal–Wallis test, *p* = 0.8861) (Table 5).

**Figure 8:**
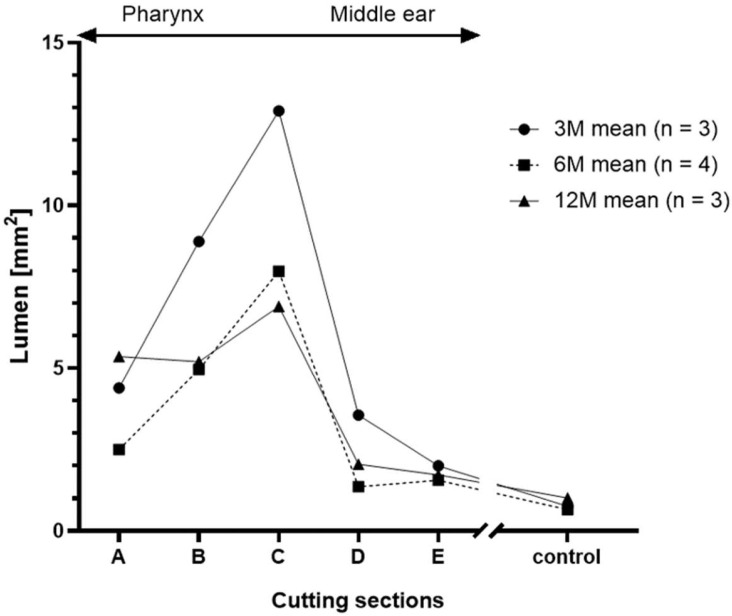
Course of the total lumen L_T_ [mm^2^] distributed over the length of the stent and the control L_C_.

Due to differences in the insertion depth, not all cases could provide slices from all defined cutting sections A–E. Section A was especially not evaluable, if stents concluded with the ET opening. However, cutting section D (containing the tapered stent end) always covered parts of the *Proc. muscularis* in all cases with stent placement in the ET (*n* = 20). The cases with full data sets (*n* = 10) of all sections A, B, C, D, and E of the histological measurements are summarized in Figure 8 showing the group mean values of the histological sections. Cutting sections supported by the stent (Wilcoxon’s matched pairs signed rank test; B: *p* = 0.002; C: *p* = 0.002; D: *p* = 0.0098) had a larger lumen (L_T_) than the associated controls (L_C_). This also applies to the sections A (*p* = 0.002) and E (*p* = 0.0039), which are not supported by the stent. Moreover, the sections A and E are typically slightly smaller than their neighboring cutting areas B and D. The analysis of variances (randomized-block) of all animals with full data sets (*n* = 10) confirmed these observations and showed significant differences with *F*(1.577, 14.19) = 20.05; *p* < 0.0001. The change in lumen between sections A to B was not significant (*p* = 0.1482), while sections B to C (*p* = 0.0183) and C to D (*p* = 0.0017) showed significant differences. Differences between sections D and E were also not significant (*p* = 0.7570). Therefore, the data show that the lumen becomes smaller at both ends of the stent, yet more to the tapered stent side. In total, these measurements demonstrated that the stent enlarges the ET lumen over the entire stent length and beyond, compared to the control.

**Figure 9:**
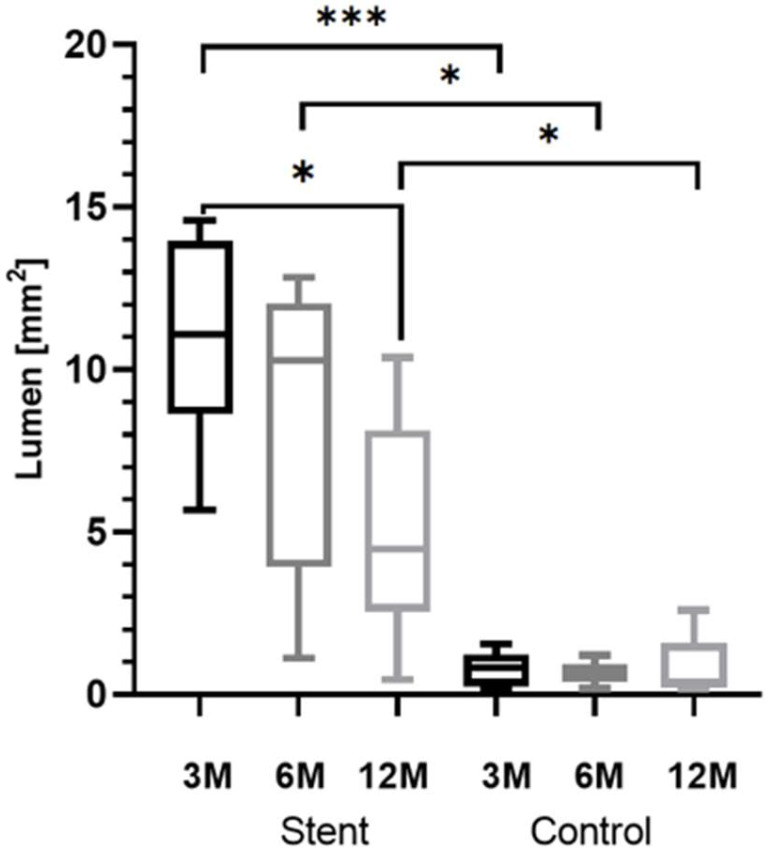
Box-and-whisker plots of the lumen L and L_C_ of all groups at section level C. Significant differences are indicated by asterisks (* *p* < 0.05; *** *p* < 0.001).

Group comparison was performed for cutting section C, which generated complete data sets for every included animal (3M: *n* = 7; 6M: *n* = 5; 12M: *n* = 6). Its positioning was corresponding to the cutting section at the control side. Paired *t*-tests showed the lumen of the stented ET was significantly larger than the lumen of the control ET for all groups (Figure 9). The results for the three groups are *t*(6) = 9.253, *p* < 0.0001 (3M), *t*(4) = 3.812, *p* < 0.0189 (6M), and *t*(5) = 3.436, *p* < 0.0185 (12M). Additionally, the stent lumen decreased over time. Analysis of variances of the stent lumen from the three groups proved that there was a significant difference *F*(2,15) = 4.1214, *p* = 0.0375. The following multiple comparisons demonstrated a significantly lower lumen for the 12M group compared to the 3M group (*p* = 0.0296) (Figure 9).

Comparing the areas covered by secretion, ANOVA did not detect any differences between the three groups with *F*(2,15) = 1.628, *p* = 0.2292. The area of the tissue (T) grown in the stent was calculated by subtracting the lumen of the stent (L) from the stent area (A_S_). Measured tissue included the mucosal epithelium and the submucosa. Microscopy indicated that the formation of granulation tissue increased over the three observation periods, which was confirmed by the reduction in lumen between 3M and 12M. However, the statistical analysis of the tissue showed no significant differences between the groups in the ANOVA with *F*(2,15) = 2.030, *p* = 0.1659. Data showed that for group 3M, 36.98% of the stent area was occupied by tissue. In the other groups, 51.43% (6M) and 69.74% (12M) of the stent area was filled with tissue, but with large variation between animals.

Furthermore, the quality of the epithelium was analyzed, and for each animal, the most frequent score of the three slices of section C was taken. The scored values of each quadrant and the control for every group were averaged (Table 5). The control ETs of all groups were always scored as category 1, indicating a good constitution of the epithelium. However, the scoring for the stented ET of the groups resulted in a shift to higher values, especially for Q4. The 6-months and 12-months group presented with an average epithelial score of 2.8 and 3.0 for Q4, respectively, which were the highest scores of all groups. Group 3M deviated from this scheme as Q1 presented with the highest score average (2.1 ± 0.7) before Q4 (1.9 ± 0.7). The comparison of the quadrants of each group with their control supported the findings. Group 3M had no significant differences (Friedman test; Q2: *p* > 0.9999; Q3: *p* > 0.9999; Q4: *p* = 0.1120), except for Q1 with *p* = 0.0352. No differences were found for group 6M (Friedman test; *p* = 0.0666). Group 12M had a significantly elevated Q4 (*p* = 0.0104), while the other quadrants did not differ from the control (Friedman test; Q1: *p* = 0.5765; Q2: *p* = 0.9413; Q3: *p* = 0.1431).

**Figure 10:**
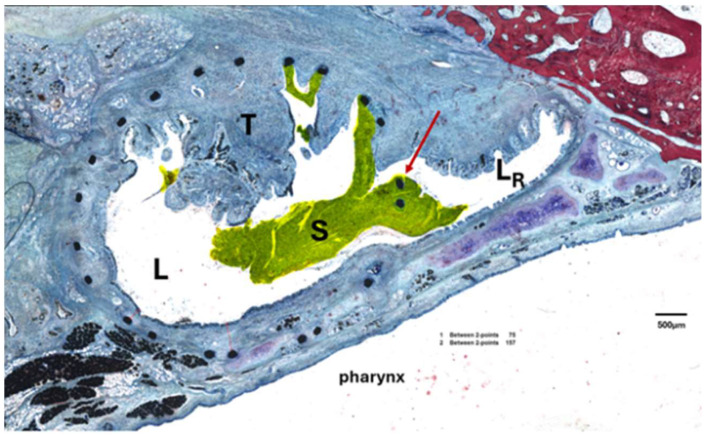
Exemplary result of the tissue reaction, if it occurred. Section B of case 6M-5 with ingrown tissue (T) and secretion (S) adhering to struts at the intersection (arrow) between stent lumen (L) and residual lumen (L_R_) in the ET.

Besides applying an epithelial score, the number of struts not covered by any tissue, and therefore laying with contact to the free lumen, was counted for each microscopic slice and averaged. As expected, they were often observed at the intersection of the stent with the residual rest of the ET lumen (Figure 10). Furthermore, if secretion was present, it was mostly associated with and/or adhering to the stent struts not overgrown by tissue. The average numbers of lumen-associated struts were *n* = 2.6 ± 1.6 for group 3M, *n* = 4.6 ± 3.8 for group 6M, and *n* = 2.3 ± 2.0 for group 12M.

### 3.6. Comparison of Results from Different Methods

If the results of the insertion process, final endoscopy, final tympanometry, CBCT, and histology data were put side by side, it became visible that these were connected and must be considered in context (compare Table 6).

The three cases with a deviating stent insertion process were the same cases with incorrect stent position in CBCT. These three stent insertions were among the first four stent insertions for that surgeon (Table 6).

In five of the nine cases, where the intended insertion depth was not reached, the ET orifice was still distinctively or severely open at the final endoscopic control or caused a fenestration. In contrast, in only three out of 15 cases with full insertion depth, an open ET orifice was detected including the two cases rated “slightly open”. Therefore, most of the stents which reached insertion depth also had closed ET orifices. In addition, six out of eight open ETs (75%) showed abnormal secretion in the final endoscopy, whereas abnormal secretion was detected in six out of 16 closed ETs (37.5%).

Animals measured with type A tympanograms (18/24) had ventilated MEs, except for 12M-2 with a partially filled (fluid and/or tissue) ME. In contrast, all animals with a final abnormal tympanogram also had abnormal ME findings. Therefore, the last tympanogram for each animal was able to detect a filled or partially filled ME in most cases (five out of six). In addition, 6M-6 had a free ME and nevertheless showed a negative middle ear pressure. However, this case had a decreased stent lumen in histology with 1.12 mm^2^ compared to the mean of the 6M group (8.43 mm^2^) (Table 5). When looking at the tympanometry data, it should be noted that the misplaced stents of 3M-4, 6M-1, and 6M-2 had no effect on the tympanograms. All three presented with mostly physiological middle ear pressure throughout the study period and all had free MEs at the end.

The animals with abnormal ME results were rather unremarkable in the endoscopy. All animals were classified with a closed or slightly open orifice. Additionally, the endoscopic secretion score was abnormal for half of the six abnormal CBCT cases (filled and partially filled), and all three were cases with completely filled MEs. The animals 6M-4 and 6M-5 had abnormal discharge in terms of character and amount and 12M-1 presented an abnormal secretion character.

Furthermore, cases with abnormal ME status were also suspicious of increased secretion areas in histology (five out of six cases) compared to the secretion of their corresponding group mean (Table 5); the exception was 12M-7 (Table 6). Group 3M stands out with eight out of eight type A tympanograms, eight out of eight free MEs, the lowest average secretion, and on average the highest lumen in histology.

## 4. Discussion

The ET marks a critical bottleneck if pathologies like mucosal inflammation, tumors, or allergies occur, or surgical procedures with effects on the ET are performed [31,41,42]. Resulting changes in the ET can lead to ETD with only limited permanent therapy options available for the patient [17].

The ET functions as a valve opening through a complex mechanism with the help of the tensor and levator veli palatini muscles [6]. A stent in the cartilaginous part could help to maintain ME ventilation if patients suffer from ETD. The aim of the current study was to investigate the long-term effects of a newly developed nitinol stent for the human ET after implantation in sheep.

Based on ethical reasons, the amount of GA per animal was limited. Therefore, no endoscopic examination of the ET during the first days (up to the individual follow-up of each group) could be performed. Endoscopic evaluations of the ET orifice shortly after implantation could have provided more information on some acute reactions to the stent.

Throughout the study, the animals tolerated the stent well without any recognizable deviations in clinical health. In addition, none of the animals showed any behavioral changes in the scoring after stent placement, suggesting that the stent did not affect their well-being. The few elevations in the score values were classified as not stent-associated. The elevated respiratory rate of 6M-8 was prior to stent insertion and the nasal discharge of 12M-3 occurred on the left nostril whereas the stent was inserted at the opposite side (right ET). Furthermore, the clinical deviations of 12M-3 could be related to endoscopy and CBCT findings. Lysis of the left nasal turbinates was diagnosed in CBCT. It is assumed that the plaque-like deposit seen before implantation was a fungus infection, which led to bone lysis and caused the associated nasal discharge with occasional snorting. Unfortunately, CBCT imaging of the full head could only be carried out after decapitation. Together with the incomplete insertion of the stent (<50%), this could have contributed to the stent loss. Therefore, it may be speculated that stent loss occurred shortly after implantation before growing tissue could have secured the stent in its position. Furthermore, 12M-3 showed recurring C-type tympanograms on both ears throughout the entire experiment. The animal reacted to tympanic measurements with a habit of swallowing prior to and during the measurements, which resulted in negative middle ear pressure. Retrospectively, this animal should have been excluded before implantation due to preexisting conditions in the nasopharynx.

The ET stent in this study was able to significantly and continuously enlarge the average ET lumen in all groups in comparison to the respective control ET of the sheep (Table 5). Recent studies have recommended a tapered design for ET stents [29,33]. The tapered shape was proven to be a good choice and seemed to provide sufficient support of the ET lumen. It was demonstrated that the expansion of the lumen followed the stent shape and decreased for the tapered end as expected. This was shown by analyzing cutting sections A–E of the stent (Figure 8). However, the results of the endoscopy show that tapering both ends could be beneficial. In addition, the rather thin mucosa of sheep ET orifices compared to humans [1,43] and stent positioning near the ET entrance could have contributed to open ETs. Thus, tapering the pharyngeal stent end could be an improvement in preventing patulous ETs.

Throughout the study, the mucosal reaction to the stent was found to be mild although no post-operative antibiotics were used to prophylactically suppress infections. As secretion was scored 0 and 1 on control sides, these values were taken as “normal” for the ET in sheep. Therefore, the secretion on the stented side was only declared as normal and abnormal after summarizing the score results. However, control ETs showed less secretion than the stented sides. Secretion did not increase during the study period. This indicated that the stent was well tolerated and only a very mild reaction in the form of secretion was detected. The endoscopic results align with the secretion measured in the histological samples as they were also found to be minor compared to the total lumen. This indicates that the drainage capacity of the ET is maintained after implantation of the current stent model. Secretion is a normal procedure of the ET, which holds cylindrical mucous-secreting cells and submucosal accessory glands [6]. This leads to the assumption that no increase in ET discharge is expected for longer observation periods than 12 months, but this still needs to be investigated. A study with an angiocatheter (1.7 × 15 to 20 mm) as stent prototype to prevent OME after nasopharyngectomy detected visible crusts, which needed to be repeatedly removed under endoscopic examination [31]. Even if the prototype was protruding into the pharynx and needed cleansing, it was more effective in preventing OME than vent tubes [31]. As a further development, the newly shaped stent used in this study was shorter and did not protrude into the pharyngeal cavity. No crusting occurred. However, in the current study, stents were inserted into healthy ETs in contrast to the study mentioned above.

Further results of this study concerning the tissue reaction must be put in relation to each other. The histological analysis proved formation of granulation tissue in the stent lumen for all groups. A visible but non-significant increase in tissue formation with observation time was present. Formation of granulation tissue is known in tracheal stents (silicone and metallic) and was the most common complication for nitinol stents [44]. Mild to moderate granulation tissue was also found in esophageal stents in a porcine model [45]. Microscopic analysis showed successful epithelization of the stent struts. This was mandatory for maintaining and regenerating the physiological clearance function of the ciliated epithelium. The epithelial score detected that an alteration of the epithelium and the lumen was obvious on the stented side, especially in quadrant 4 compared to the control (Table 5). It should be noted that quadrants 3 and 4 were facing the lateral side of the ET. Q3 and Q4 were exposed to the highest amount of movement forced by the connected muscles during natural opening attempts of the ET (see Figure 3). More formation of tissue was present in the stent lumen in these areas. It is concluded that, physiologically, more movement of the ET affects the stent and could have induced the development of tissue and incomplete epithelium. Tissue ingrowth occurred, yet the stent lumen remained significantly larger than in the control lumen for all groups. Even if tissue ingrowth into the lumen was detected, there was in general enough epithelial lining present. The ciliary activity of the cells seemed sufficient since secretion was transported to the pharynx, and both the secretion score and the determined secretion area in the lumen were only small. However, some fluid-filled MEs were detected, indicating that clearance was not sufficient in all animals. Reasons for this remain unknown. A recent study showed that tapered ET stents were superior to tubular stents in terms of tissue hyperplasia [33]. Furthermore, drug-eluting stent designs could decrease the formation of granulation tissue. Drug-eluting ET stents were only tested in a porcine model with very small numbers [46]. The sirolimus-covered stents showed a promising reduction of tissue hyperplasia [46]. In addition, steroid-eluting (mometasone furoate) sinus stents were already used in humans and improved the surgical outcome in patients [47]. Hence, it is reasonable that a coating could have limited or even prevented the tissue growth that occurred in the current study.

Visualization (Figure 8) of the histological results of samples with complete cutting sections A–E showed the stent increased the lumen beyond the stent ends (section A and E) compared to the controls. However, section B had a smaller lumen than section C even if both were of the same diameter (5 mm). Consequently, more tissue ingrowth occurred in section B compared to C. This can be related to the transition zone from mucosa to stent-supported mucosa in section B. Other studies showed that formation of granulation tissue often occurred at the stent ends. Granuloma formation at the stent ends of a self-expanding tracheal (nitinol) stent was detected in three out of 26 cases [48]. Furthermore, increased granulation tissue was found at esophageal stent ends (nitinol) in a porcine model [45].

Additionally, the individual insertion process influenced the study outcome of each animal. In some cases, surgeons reported feelings of resistance during the insertion of the tool. Consequently, the intended insertion depth seemed to be insufficient in several cases (Table 6). The general variance of the individual sheep anatomy and thus the size of the sheep’s head could have been a reason for that. Consequently, different tool angles were needed to insert the application tool into the ET. In addition, four stents (3M-2, 6M-8, 12M-6, and 12M-7) were slightly retracted whilst removing the tool from the ET lumen. This issue was considered the cause for the perforated and fenestrated ET (case 6M-8) and could have contributed to patulous ETs. The anatomy of the *O. pharyngeum* of sheep is different compared to humans [43]. Humans have a mucosal protrusion, the *Torus tubarius*, strengthening the ET opening. For this reason, perforation of the stent is not expected for human application. However, the above-discussed tapering of the stent at its pharyngeal end would also help to avoid perforation of the medial tubal walls.

Moreover, initial insertion characteristics were still traceable throughout the experiment. Visible stents in the first follow-up were very likely to remain visible in the next follow-ups as stents did not migrate if initially ingrown into the mucosa. This also applies to deposits at the ET orifice, which were likely to be detected in both follow-ups if they occurred. These deposits were suspected to contain mucus with inflammatory cells. In general, stents showed no migration, and the location, if visible, was consistent in all endoscopic examinations. The deviating stent insertion process of 3M-4, 6M-1, and 6M-2 was also traceable in the further course of the experiment. These stents were the same three stents, which were found to be misplaced in CBCT. Retrospectively, the insertion process was not carried out as intended. Video endoscopy revealed that the tool was slightly pushed forward instead of only retracting the outer sheath to release the stent. This made the releasing process rather active than passive and resulted in pushing the stent into the surrounding tissue. It is worth mentioning that this error occurred during the first stent insertions (first, second, and fourth) by this surgeon despite training on cadaver heads. All other stents were placed correctly. This highlights the need for more intensive training for handling and application of the stent tool for future studies. Fortunately, the incorrect placement had no negative effects on animals’ health or the tympanometry and CBCT outcomes. These animals had mostly physiological tympanograms throughout the study, and all had free MEs.

CBCT proved that no stent dislocation occurred except for the animal (12M-3) with the affected turbinates. All were found at the expected location except for the three misplaced ones. In other studies, stent migration was a known complication. Silicone especially, but also metallic stents (stainless steel and covered nitinol), were found to be dislocated after airway stenting in follow-ups (median 41.4 months) of 100 children [44]. Esophageal SEMS did not migrate in a porcine model [45]. The results of the current study underline the safety of the application process as no stent migration into sensitive areas of the head is to be expected, even when stents are misplaced. This is important, especially due to the proximity of the ET to the inner carotid artery (ICA), e.g., it was described that BET might be a risk for dissected ICAs [49]. In addition, CBCT revealed that insertion depth was mostly reached, even if the endoscopic score applied to the insertion process indicated otherwise. This was also evident in the histological analysis. Slices of the stent section D from all correct stent placements (20/24) showed the presence of the *Proc. muscularis* (Figure 6). Therefore, stent positioning in most cases was as deep as the individual anatomy would have allowed, except for the stents, which were retracted to the pharyngeal orifice while the tool was removed from the ET. This concludes that the insertion depth evaluated by endoscopy is questionable, and CBCT results should be considered for evaluating the stent position. Nevertheless, the importance of also reaching the intended insertion depth under endoscopic observation is underlined by the fact that the risk of generating a patulous ET was much higher when the intended insertion depth was not met. In human application, CBCT data would be available before stent insertion to select the adequate stent dimensions to fit the individual anatomy of a patient. Thus, a patulous ET could be avoided. In addition, the tool measures were designed to not pass the ET isthmus region into the ME. This was achieved as no stent passed the isthmus and protruded into the ME. Another study also described the importance of this, as an ET stent was accidentally pushed through the isthmus up to the ME, releasing the stent with proportions in the ME [28]. Our study showed the feasibility of a stent application with no risk of insertion and release into the isthmus or even further into the ME. All stents were placed as desired into the cartilaginous portion if the releasing process was conducted correctly. Nevertheless, in further studies, a possibility to integrate repeated in vivo CBCT imaging also in sheep would be advantageous.

Sheep are ruminants and therefore chew and swallow more than humans. Fujihara et al. showed that sheep chewing whole hay spend more than 7 h (427.2 ± 36.0 min) per day chewing [50]. For this reason, ET stents were exposed to an increased mechanical stress in sheep. However, CBCT showed intact stents and no stent fractures occurred. In humans, the ET opens on average one to two times per minute [51]. It can therefore be assumed that the mechanical load on the stent is probably lower in humans than in sheep.

Results from CBCT also matched the tympanometry outcome. All animals with type B tympanograms at the last measurement (five out of 24) had filled or partially filled MEs in CBCT. Thus, tympanometry effectively predicted the abnormal MEs. Additionally, initial disturbances of the ME ventilation in week 1 post-stent insertion were detected in the tympanograms. Animals developed type C tympanograms and, in some cases, transitioned to type B tympanograms, which did not recover. A presumable cause could be a mucosal swelling and/or epithelial decline due to the insertion process. This assumption was supported by a study on BET and its histological findings in 20 rats [52]. The ET lumen initially increased after BET but decreased at week 1 due to increased submucosa depth and epithelial hyperplasia. The lumen recovered to normal after 4 weeks and the epithelium fully recovered after 12 weeks post-BET [52]. Therefore, the first week seems to be a critical time and, again, antiproliferative coatings could help to prevent and/or attenuate tympanogram deviations in the first weeks after stent implantation.

In addition, sporadic, recurring type C tympanograms occurred on both ears and in one case were related to excessive swallowing (12M-3). This confirmed that recurring and non-constant type C tympanograms were a normal incident in sheep tympanometry [35]. Type C tympanograms should only be considered problematic if they persist or are followed by type B, which could indicate OME. Besides, the individual tympanogram results of each animal had a major influence on the outcome of the group results as there were isolated animals with constant type B tympanograms, whereas some were always measured with physiological middle ear pressure (see Appendix A). Overall, tympanometry was an essential monitoring tool for examining the ET stents in vivo. It was the only direct evidence of middle ear health and thus also indirect verification of ET function. It is therefore reasonable to assume that all MEs with physiological tympanograms (stented and control sides) have Eustachian tubes with sufficient patency to provide adequate ventilation to the middle ear. Animals with type B or type C tympanograms and/or filled MEs did not have adequate patency, although histology showed a total lumen in section C in all animals. Consequently, tympanometry should be considered for evaluation of ET stents. Furthermore, the order of stent implantations could have influenced the results. It was striking that the tympanometry results of group 3M, which were predominantly implanted later in the trial, were better (see Table 6). This is especially true as due to weekly measurements in all groups, results could be directly compared between groups. Group 12M was predominantly implanted early due to the long observation period of this trial group.

## 5. Conclusions

Implantation of a tapered ET stent made from nitinol appears to be safe if the insertion procedure is performed correctly. Training of the insertion procedure is necessary, and the stent can reliably be positioned in the cartilaginous part of the ET. Using the correct stent dimensions for the individual case enables stenting the ET without generating a patulous ET. The open lumen of the ET is enlarged even after 12 months of implantation and the epithelium layer can regenerate. As the lumen of stented ETs was in all groups larger than in controls without stent, long-term implantation of the tested stent appears feasible.

## Figures and Tables

**Table 1:** Insertion depth of application tool.

Insertion Depth	Group 3M	Group 6M	Group 12M
100%	4	7	4
>50%	2	1	1
<50%	2	0	3

Provided are numbers of animals.

**Table 2:** Degree of opening of the pharyngeal ET orifice at the three observation points for each group.

**Group 3M**	**Day 1**	**1 ½ Months**	**3 Months**
Closed	3	2	3
Slightly open	1	2	1
Distinctively open	3	1	1
Severely open	1	3	3
**Group 6M**	**Day 1**	**3 Months**	**6 Months**
Closed	4	4	6
Slightly open	3	2	/
Distinctively open	1	1	/
Severely open	/	1	2
**Group 12M**	**Day 1**	**6 Months**	**12 Months**
Closed	2	6	7
Slightly open	3	2	1
Distinctively open	2	/	/
Severely open	1	/	/

Provided are numbers of animals with the different opening degrees.

**Table 3:** Summary of tympanometry results of the stented and control ear for each group [%].

Group	Stent/Control		
	Type A	Type B	Type C
3M	93.75%/80.21%	2.08%/12.50%	4.17%/7.29%
6M	55.77%/84.13%	27.88%/13.94%	16.35%/1.92%
12M	61.06%/91.35%	30.77%/0.24%	8.17%/8.41%

**Table 4:** Measured diameters [mm] of the three stent cross-sections.

Group	Stent Section	Largest Diameter	Orthogonal Diameter
3M (*n* = 8)	d1	3.1 ± 0.1	3.0 ± 0.1
	d2	5.1 ± 0.1	4.9 ± 0.1
	d3	5.1 ± 0.2	4.9 ± 0.2
6M (*n* = 7)	d1	3.1 ± 0.1	2.9 ± 0.1
	d2	5.1 ± 0.2	4.9 ± 0.1
	d3	5.0 ± 0.1	4.9 ± 0.1
12M (*n* = 7)	d1	3.2 ± 0.2	2.9 ± 0.2
	d2	5.1 ± 0.2	4.9 ± 0.2
	d3	5.1 ± 0.3	4.8 ± 0.4

Data are shown as mean ± SD; 6M is listed without the compressed stent 6M-2.

**Table 5:** Areas and epithelial score for the stented and control ET sides (cutting section C).

Control		L_C_		S_C_			Epithelial Score			
3M		0.76 ± 0.52		0.07 ± 0.03			1.0 ± 0.0			
6M		0.64 ± 0.37		0.11 ± 0.08			1.0 ± 0.0			
12M		0.83 ± 0.95		0.09 ± 0.07			1.0 ± 0.0			
**Stent**	**A_S_**	**L_T_**	**L**	**S**	**T**	**S_R_**	**Q1**	**Q2**	**Q3**	**Q4**
3M	17.78 ± 1.28	11.86 ± 3.56	11.05 ± 3.24	1.22 ± 1.22	6.73 ± 4.16	0.13 ± 0.11	2.1 ± 0.7	1.4 ± 0.8	1.3 ± 0.5	1.9 ± 0.7
6M	17.91 ± 1.22	9.02 ± 4.97	8.43 ± 4.67	3.11 ± 3.33	9.48 ± 5.85	0.22 ± 0.25	1.4 ± 0.9	2.0 ± 0.7	2.4 ± 1.3	2.8 ± 1.1
12M	17.19 ± 2.85	6.10 ± 4.35	5.07 ± 3.47	1.32 ± 0.83	12.12 ± 4.65	0.27 ± 0.20	1.7 ± 0.8	1.7 ± 0.5	2.5 ± 1.2	3.0 ± 1.1

Mean ± SD area measurements [mm^2^] and epithelial score for the quadrants (Q) 1–4 for each group (3M: *n* = 7; 6M: *n* = 5; 12M: *n* = 6). L_C_—lumen of the control; S_C_—area of secretion (control); A_S_—area of the stent; L_T_—area of the total ET lumen; L—area of the lumen within the stent; S—area of L taken by secretion; T—area of A_S_ occupied by tissue; S_R_—area of secretion outside the stent lumen.

**Table 6:** Summary of implantation-related data and results collected at the end of the observation periods.

	Stent Insertion				Endoscopy			Tymp	CBCT	Histology	
Case ID	Surgeon	Order of Implantation	Insertion Angle	Insertion Process	Insertion Depth	Final Opening Grade	Final Secretion	Final Curve Type	ME Status	Secretion [mm^2^]	Lumen [mm^2^]
3M-1	2	21	20°	correct	>50%	open ^3^	abnormal ^D^	A	free	0.22	11.08
3M-2	2	22	25°	correct	100%	closed	abnormal	A	free	0.36	13.53
3M-3	2	5	20°	correct	<50%	open ^3^	abnormal ^D^	A	free	3.74	9.91
3M-4	2	6	10°	incorrect	100%	closed	normal	A	free ^M^	/	/
3M-5	2	15	30°	correct	100%	closed	normal	A	free	1.20	8.63
3M-6	2	16	25°	correct	>50%	open ^2^	abnormal ^D^	A	free	1.44	5.67
3M-7	2	23	25°	correct	<50%	open ^3, H^	normal ^D^	A	free	0.33	14.59
3M-8	2	24	30°	correct	100%	open ^1, H^	normal	A	free	1.26	13.95
6M-1	2	4	45°	incorrect	100%	closed	normal	A	free ^M^	/	/
6M-2	2	3	0°	incorrect	100%	closed	normal	A	free ^M^	/	/
6M-3	2	17	30°	correct	100%	open ^3^	abnormal	A	free	0.23	12.84
6M-4	2	18	25°	correct	100%	closed	abnormal	B	filled	6.49	10.28
6M-5	2	11	20°	correct	100%	closed	abnormal	B	filled	6.93	11.23
6M-6	2	12	30°	correct	100%	closed	normal	C	free	0.30	1.12
6M-7	2	19	30°	correct	100%	closed	abnormal	A	free	1.61	6.69
6M-8	2	20	30°	correct	>50%	open ^F^	abnormal ^D^	A	free	/	/
12M-1	1	1	45°	correct	100%	open ^1^	abnormal	B	filled	1.79	10.37
12M-2	1	2	45°	correct	100%	closed	normal	A **	partially filled	1.54	7.38
12M-3	2	8	10°	correct	<50%	closed	normal	A	free ^L^	/	/
12M-4	2	7	20°	correct	>50%	closed	abnormal	A	free	/	/
12M-5	2	13	20°	correct	<50%	closed	normal	A	free	0.33	0.46
12M-6	2	14	15°	correct	<50%	closed	abnormal	A	free	0.91	3.56
12M-7	2	9	20°	correct	100%	closed	normal ^D^	B *	filled	0.71	5.39
12M-8	2	10	20°	correct	100%	closed	normal	B	partially filled	2.62	3.26

Tymp—tympanometry; superscript annotations include the following: 1—slightly open, 2—distinctively open, 3—severely open, H—a blade of hay intruding the ET lumen, and F—fenestration; D—deposits at the ET orifice; *—scarring at the tympanic membrane (TM); **—alterations at the TM prior to stent insertion until final follow-up; M—misplaced stents visible in CBCT; L—lost stent.

## Data Availability

The data, which led to the results of this study, are available on request from the corresponding authors.

## Appendix A

Tympanometry data of the three groups showing allocated curve types for each week over the observation period for the stent and control sides.
